# Gaining insights into virotherapy with canine models

**DOI:** 10.1016/j.omto.2023.100754

**Published:** 2023-12-02

**Authors:** Jacob L. Léger, Lee-Hwa Tai

**Affiliations:** 1 Department of Immunology and Cell Biology, Université de Sherbrooke, Sherbrooke, QC J1E 4K8, Canada; 2 Research Centre of the Centre Hospitalier de l’Université de Sherbrooke (CRCHUS), Sherbrooke, QC J1H 5N4, Canada

The field of oncolytic viruses (OVs) is rapidly gaining traction in clinical oncology, with the increasing interest in leveraging OVs as both standalone therapies and in combination with existing approved treatments. As this therapeutic landscape evolves, a deeper understanding of the underlying biological mechanisms and pharmacokinetics of OVs becomes imperative. The study by Makielski and colleagues, featured in the current *Molecular Therapy – Oncolytics* issue, delves into the potential of neoadjuvant systemic OV therapy. Specifically, they explore the application of a recombinant oncolytic vesicular stomatitis virus (VSV) expressing interferon-beta (IFNβ) and the sodium iodide symporter (NIS) VSV-IFNβ-NIS in the context of osteosarcoma in companion dogs.^1^

Osteosarcoma, a devastating bone cancer primarily afflicting the pediatric and young adult populations, continues to present challenges in managing metastatic disease progression despite the conventional therapeutic approaches of surgical resection and chemotherapy.^2^ Against this backdrop, Makielski and colleagues explore the potential of neoadjuvant VSV-IFNβ-NIS therapy, a strategy that involves administering the OV before standard-of-care surgical resection. This innovative approach not only sets the study apart but it also opens new avenues for investigation into OV as a novel component in the arsenal against solid tumors.^3^

The methodology used in the study is noteworthy for its comprehensive nature. Administering neoadjuvant VSV-IFNβ-NIS allowed for the careful microscopic and genomic analysis of tumors. This dual approach enabled a multifaceted exploration of the impact of the OV on primary and metastatic tumor sites. The meticulousness of this analysis provides a robust foundation for the study’s findings and contributes to the depth of understanding of the complex interactions between the OV and the tumor immune microenvironment.^4^

A distinctive strength of this study lies in its use of companion dogs with naturally occurring osteosarcoma. Canine osteosarcoma is recognized as a suitable model for human osteosarcoma due to similarities in natural disease history. This translational relevance enhances the significance of the study’s findings, bridging the gap between veterinary and human medicine. It not only broadens the applicability of the research but it also lays the groundwork for potential advancements in both realms of cancer treatment.^5^

The standout results include the well-tolerated nature of the treatment, a significant proportion of long-term survivors in the VSV-treated group, and an increase in tumor inflammation. Of particular note is the enhanced expression of a T cell–anchored immune gene cluster in all of the long-term responders, indicating a robust and sustained antitumor immune response. These promising outcomes validate the potential efficacy of neoadjuvant VSV-IFNβ-NIS therapy and provide hope for improved long-term clinical outcomes in human osteosarcoma patients.^5^

Looking beyond the immediate findings of the study opens doors to broader perspectives on the future of virotherapy. The demonstrated potential of OVs to induce antitumor immune responses prompts future investigations into tailoring OVs to individual patients based on their individualized immune profiles and preexisting immunity. In addition, the identified T cell–focused immune gene cluster in long-term responders presents opportunities for synergistic combinations with various immunomodulatory agents.

Although traditional immune checkpoint inhibitors are under study in combination with OVs, emerging immunotherapies such as chimeric antigen receptor T cells and tumor-infiltrating lymphocyte therapy also warrant exploration. This points toward a dynamic future in which the marriage of different modalities could enhance the efficacy of virotherapy, offering more personalized and effective treatment strategies. Furthermore, the study hints at the possibility of using OVs to address resistance mechanisms, including those associated with immune checkpoint inhibitors. Future OV research could delve into the underlying mechanisms of immune checkpoint inhibitor resistance and resistance to other therapies. This presents fertile ground for developing focused strategies to augment the efficacy of OVs in overcoming these challenges, potentially broadening the applicability of virotherapy in diverse clinical scenarios.^6^

Although the study’s findings are promising in the canine model, translating these results to human osteosarcoma patients and patients with other solid tumors requires further investigation. Relatively lower genetic heterogeneity within the canine population as compared to human populations limits the generalizability of these findings. Ultimately, testing oncolytic VSV-IFNβ-NIS with human patients is necessary to validate its efficacy and safety.

In conclusion, Makielski and colleagues have made a significant advancement in unraveling the potential of neoadjuvant VSV-IFNβ-NIS therapy in osteosarcoma. Although recognizing and praising the strengths of the study, including its innovative methodology, comprehensive analysis, and promising clinical outcomes, it is crucial to acknowledge its limitations. The path forward involves addressing these limitations through continued preclinical, translational, and clinical research endeavors. The perspective on OV therapy emphasizes the ongoing need for exploration, combination strategies, understanding mechanisms of action, safety profiles, and the critical translation into human patients to maximize its transformative impact on cancer treatment.
